# Renal insufficiency retains adverse prognostic implications despite renal function improvement following Total Therapy for newly diagnosed multiple myeloma

**DOI:** 10.1038/leu.2015.15

**Published:** 2015-03-13

**Authors:** R Khan, S Apewokin, M Grazziutti, S Yaccoby, J Epstein, F van Rhee, A Rosenthal, S Waheed, S Usmani, S Atrash, S Kumar, A Hoering, J Crowley, J D Shaughnessy, B Barlogie

**Affiliations:** 1 Myeloma Institute for Research and Therapy, University of Arkansas for Medical Sciences, Little Rock, AR, USA; 2 Cancer Research and Biostatistics, Seattle, WA, USA; 3 Levine Cancer Institute/Carolinas Healthcare System, Charlotte, NC, USA; 4 Signal Genetics Inc, Carlsbad, CA, USA

## Abstract

Renal insufficiency (RI) is a frequent complication of multiple myeloma (MM) with negative consequences for patient survival. The improved clinical outcome with successive Total Therapy (TT) protocols was limited to patients without RI. We therefore performed a retrospective analysis of overall survival, progression-free survival and time to progression (TTP) of patients enrolled in TT2 and TT3 in relationship to RI present at baseline and pre-transplant. Glomerular filtration rate was graded in four renal classes (RCs), RC1–RC4 (RC1 ⩾90 ml/min/1.73 m^2^, RC2 60–89 ml/min/1.73 m^2^, RC3 30–59 ml/min/1.73 m^2^ and RC4 <30 ml/min/1.73 m^2^). RC1–3 had comparable clinical outcomes while RC4 was deleterious, even after improvement to better RC after transplant. Among the 85% of patients with gene expression profiling defined low-risk MM, Cox regression modeling of baseline and pre-transplant features, which also took into consideration RC improvement and MM complete response (CR), identified the presence of metaphase cytogenetic abnormalities and baseline RC4 as independent variables linked to inferior TTP post-transplant, while MM CR reduced the risk of progression and TTP by more than 60%. Failure to improve clinical outcomes despite RI improvement suggested MM-related causes. Although distinguishing RC4 from RC<4, 46 gene probes bore no apparent relationship to MM biology or survival.

## Introduction

Renal insufficiency (RI) is a common complication of multiple myeloma (MM) that can be present at diagnosis or emerge during therapy^1, 2^ and represents a feature of Cancer Research and Biostatistics criteria constituting the need for instituting MM therapy.^3^ The etiology of RI is often multifactorial; hypercalcemia and any of the myeloma-protein-associated conditions such as light-chain cast nephropathy, light-chain amyloidosis and light-chain deposition disease are common causes. Hypercalcemia and light-chain cast nephropathy readily respond to hydration and effective myeloma therapy.^4, 5, 6^ In case of high tumor burden and high-grade characteristics, effective treatment can provoke tumor lysis and thus cause renal shut-down.^7^ Additionally, nephrotoxic antibiotics and bisphosphonates can aggravate or cause renal impairment. The intricate interplay between renal function and MM is complex and of interest because RI has important implications for survival. The adverse survival consequences of RI have long been acknowledged,^2, 8^ accounting for the B sub-stage designation in the Durie-Salmon staging system.^9^ RI as a prognosticator for survival has been retained indirectly in the albumin- and β-2-microglobulin (B2M)-based International Staging System.^10^ The B2M molecule is shed from the surface of MM cells so that its serum levels reflect tumor burden,^11^ but due to the renal excretion of B2M, RI can further raise B2M serum levels.^12^ Poor clinical outcomes resulting from RI are usually attributed to higher treatment-related mortality.^12, 13, 14, 15, 16, 17^

The introduction of bortezomib has greatly improved survival outcomes in MM, both in transplant and non-transplant settings. When bortezomib, not requiring adjustment for renal function, was added to melphalan-prednisone in the VISTA trial, RI was not an adverse feature in the experimental arm.^18^ In the HOVON-65/GMMG-HD4 trial, the prognostic impact of RI was investigated in two treatment arms, both including melphalan-based auto-transplants.^13^ One arm received bortezomib, adriamycin and dexamethasone (PAD) induction prior to and bortezomib maintenance after autologous stem cell transplant, while the other arm was given vincristine, adriamycin and dexamethasone (VAD) induction and thalidomide maintenance. Although renal response rates were similar after PAD and VAD, MM response rates including complete response were higher with PAD, as were overall survival (OS) and progression-free survival (PFS). In fact, OS and PFS were independent of RI in the PAD arm and resembled outcomes of patients without RI treated with VAD. The observation of similar renal responses to VAD and PAD and yet inferior survival with VAD suggested a RI-associated adverse MM feature that could be overcome by the inclusion of bortezomib in PAD.

Reviewing clinical outcomes after successive Total Therapy (TT) trials, significant survival advances were observed with the transition from TT1 to TT2 and TT3.^19^ The incorporation of bortezomib into induction, consolidation and maintenance phases of TT3 led to dramatic improvement in clinical outcomes that, however, was limited to the 85% of patients with plasma cell gene expression profiling (GEP)-defined low risk. The 15% with high-risk disease (GEP70>0.66) continued to fare poorly despite the addition of bortezomib and immune-modulatory agents. We also observed that there was a lack of progress in the transition from TT2 to TT3 in the case of RI. We now examine whether the lack of clinical outcome improvement was limited to patients who did not improve from baseline RI.

## Materials and methods

Records were reviewed from our data base of all MM patients (*N*=1148) enrolled and followed in TT2 without thalidomide (TT2−Thal; *n*=345), TT2 with thalidomide (TT2+Thal; *n*=323), and TT3 (*n*=480) between 14 October 1998 and 29 January 2014. Details of these protocols and patient outcomes have previously been reported.^20, 21, 22, 23^ TT3b (*n*=177) differed from TT3a (*n*=303) only in the maintenance phase such that in TT3b bortezomib, lenalidomide and dexamethasone was applied for all 3 years whereas TT3a employed bortezomib, thalidomide and dexamethasone only in the first year and subsequently only thalidomide and dexamethasone. In TT2, RI was not an exclusion criterion as long as it was of recent onset (<2 months) and due to Bence Jones proteinuria or hypercalcemia. Cisplatin dosing in cycle 2 with dexamethasone, cyclophosphamide, etoposide and cisplatin was modified according to severity of RI. Patients with serum creatinine ⩽1.5 mg/dl received 15 mg/m^2^; cisplatin dosing was reduced to 10 mg/m^2^ for creatinine of 1.6–2.0 mg/m^2^ and to 7.5 mg/m^2^ for creatinine 2.1–3.0 mg/dl, while it was omitted in case of creatinine >3.0 mg/m^2^. TT2 also restricted melphalan dosing to 140 mg/m^2^ during the transplant phase when serum creatinine was ⩾3.0 mg/dl. TT3 patients were eligible when serum creatinine values did not exceed 3 mg/dl. Induction cisplatin was modified from the full dose of 10 mg/m^2^ to 5 mg/m^2^ for serum creatinine of 1.6–2 mg/dl and drug was omitted with creatinine >2 mg/dl. As in TT2, melphalan dosing in the transplant phase was reduced to 140 mg/m^2^ for creatinine levels of ⩾3.0 mg/dl. GEP risk designation was applied as previously reported.^24^

Clinical outcome data included OS and PFS. We also examined time to progression (TTP). For the purpose of this analysis, we calculated the estimated glomerular filtration rate (eGFR) for all patients, using the original Modification of Diet in Renal Disease equation^25^ as recommended by International Myeloma Working Group.^5^ The aforementioned clinical end points were examined among four renal classes (RCs): RC1 (eGFR ⩾90 ml/min/1.73 m^2^), RC2 (eGFR 60–89 ml/min/1.73 m^2^), RC3 (eGFR 30–59 ml/min/1.73 m^2^) and RC4 (eGFR <30 ml/min/1.73 m^2^).

All protocols had been approved by the University of Arkansas Medical Sciences Institutional Review Board. Patients were required to sign a written informed consent in keeping with institutional, federal and the Declaration of Helsinki guidelines. Annual Data Safety and Monitoring Board and semi-annual external auditor reviews were also performed according to National Institutes of Health mandates for federally supported research grants.

### Statistical analyses

Univariate baseline characteristics were compared between protocols using *χ*^2^-tests. RC comparisons were made between RC1-3 and RC4, since OS and PFS were similar between RC1, RC2 and RC3, while RC4 predicted uniquely poor OS and PFS. Kaplan–Meier curves were compared using the log-rank test.^26^ TTP was analyzed by estimating the cumulative incidence of the given outcome,^27^ and compared between RC groups (1–3 vs 4) by the log-rank test. Cox proportional hazards regression models were employed to identify associations between RC groups (1–3 vs 4) and survival outcomes.^28^ Multivariate models were arrived at using stepwise model selection with entry level *P*-value of 0.1, where the variable remained in the model if it was significant at the 0.05 level. An indicator of TT3 protocol was included in the multivariate analyses to account for the use of bortezomib in TT3a and TT3b compared with TT2 regimens. Employing a false discovery rate of *q*=0.05, 46 significant gene probes were identified that distinguished these RI subsets.^29^

## Results

**Figure 1:**
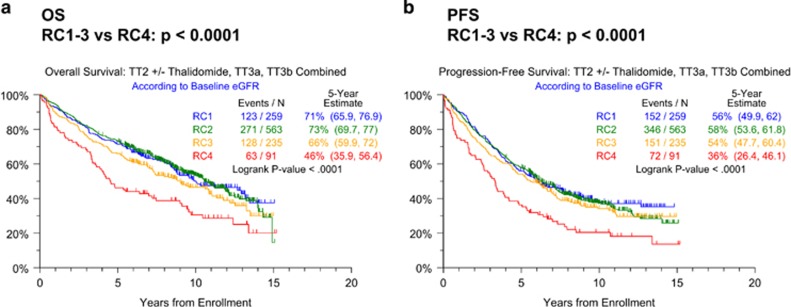
Clinical outcomes with Total Therapy protocols (TT2, TT3) according to baseline eGFR class (RC1-4). (**a**) Overall survival; (**b**) progression-free survival.

**Figure 2:**
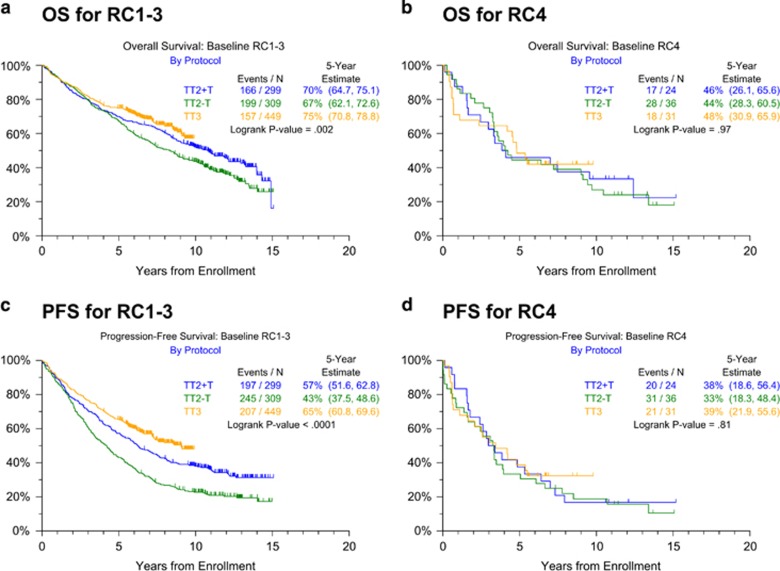
Overall and progression-free survival by Total Therapy protocol (TT2–Thal, TT2+Thal, TT3a and TT3b combined) for RC1-3 and RC4 baseline classes. (**a**) Overall survival for RC1–3; (**b**) overall survival for RC4; (**c**) progression-free survival for RC1-3; and (**d**) progression-free survival for RC4.

Patients' baseline characteristics including RC distributions were largely similar across the treatment regimens. TT3 comprised higher proportions of patients with high B2M levels >5.5 mg/l, hypo-albuminemia <3.5 g/dl and Bence Jones proteinuria (Table 1). OS and PFS are depicted across all protocols according to the baseline RC (Figure 1). Clinical outcomes in patients with RC1–3 bundled together, while RC4 was associated with inferior OS (Figure 1a) and PFS (Figure 1b). Given the strikingly different outcomes between patients in RC1–3 versus RC4 classes, we next examined the outcomes in these two RC groups by protocol (Figure 2). Both OS and PFS improved markedly with the transition from TT2−Thal to TT2+Thal to TT3 in patients with RC1–3 (Figures 2a and c), however, such progress was not apparent in RC4 (Figures 2b and d). One possible explanation might be that RC4 patients could not be treated in as timely a fashion as their RC1–3 counterparts. However, the succession through protocol phases was similar in RC4 and RC1–3 groups across protocols (data not shown). Examining PFS in order to capture both progressions and deaths, we employed Cox-regression analysis with all relevant baseline variables. The univariately adverse effect of RC4 (hazards ratio=1.73, *P*<0.001) was not retained after adjustment for other parameters, whether or not GEP70 risk was considered (Tables 2A and 2B). In fact, well-recognized standard features like low albumin, elevated serum levels of B2M and lactate dehydrogenase, cytogenetic abnormalities (CA), low platelet count and immunoglobulin A isotype all imparted shorter PFS, joined by GEP70 high risk when this variable was included (Table 2B); TT3 reduced the hazard of progression by about 40%. When limited to GEP70 low-risk MM, RC4 remained a strong adverse feature after adjusting for the other parameters (Table 2C).

**Figure 3:**
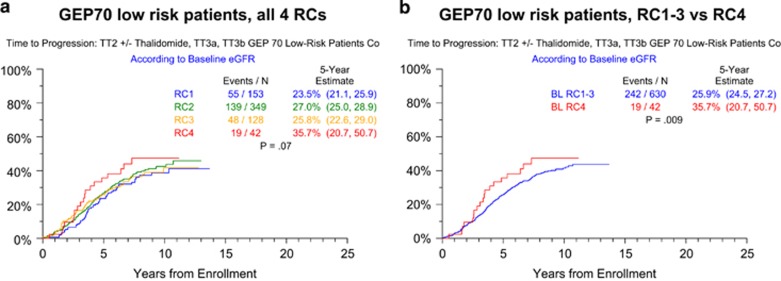
Time to progression in GEP70-defined low-risk myeloma according to RC. (**a**) By individual RC quartiles; (**b**) RC4 versus RC1-3.

Subsequent analyses concentrated on the GEP70 low-risk cohort, in order to focus on the population for which RC4 imparts poorer outcomes relative to RC1–3. To emphasize MM-related events, we investigated TTP in GEP70-defined low-risk MM (Figure 3). The significantly steeper onset of progression in RC4 compared with RC1–3 patients suggests that the difference in survival is MM related. This observation prompted us to investigate whether, in patients with GEP70 low-risk MM, there were RC-dependent differences in baseline characteristics. Indeed, although the frequency of CA overall was similar between RC groups (41% with RC4 vs 31% with RC1–3; *P*=0.07), high-risk CA types especially CA13 and/or hypodiploidy were overrepresented in RC4 (40% vs 19% in RC<4; *P*<0.001). Logistic regression analysis that excluded creatinine and B2M for their known correlations with eGFR revealed that high C-reactive protein (⩾8 mg/l, hemoglobin <10 g/dl, high lactate dehydrogenase (⩾190 U/l) and bone marrow plasmacytosis (⩾33%) were all independently linked to RC4 (Table 3).

Then we examined GEP signatures among GEP70-defined low-risk MM relative to RC4 and RC<4 groups. Employing a false discovery rate of *q*=0.05, 46 significant gene probes were identified that distinguished these RI subsets (Supplementary Tables 1A and 1B). We failed to recognize a plausible relationship of the listed gene probes to MM biology, and an impact on survival was not apparent.

**Figure 4:**
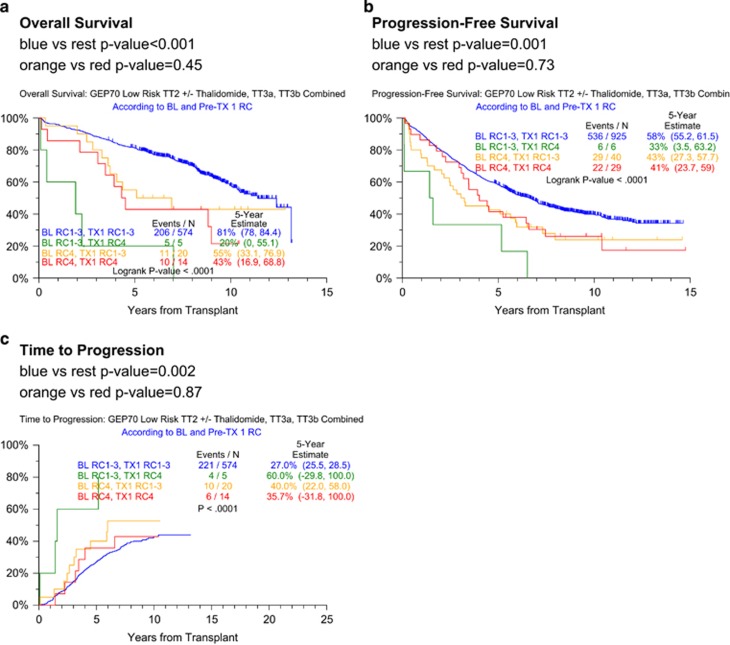
Overall survival, progression-free survival and time to progression in GEP70-defined low-risk myeloma according to baseline and transplant RCs. (**a**) Overall survival is superior with RC1–3 at both time points; (**b**) progression-free survival is superior with RC1–3 at both time points; (**c**) time to progression most shallow with RC1–3 at both time points.

In the following section, we analyze the effect of RC improvement after induction therapy on post-transplant outcomes in low-risk MM (Figure 4). Both OS and PFS were superior when RC1–3 status was maintained pre-transplant (Figures 4a and b). The gravest outcome applied to patients who moved from RC1–3 at baseline to RC4 at transplant, although there are very few patients in this group. The remaining patients (RC4 at both time points, RC4 improving to RC1–3) had an intermediate outcome. The same directional effect applied to TTP (Figure 4c). Next, we investigated whether our GEP46 model could distinguish, at baseline, the RC4 to RC1–3 converts from patients retaining RC4 status. Results revealed, possibly due to small sample size, no baseline differences between these two groups (data not shown).

In order to account for baseline and pre-transplant variables collectively in the context of RC change and clinical response, a further multivariate analysis examining PFS and TTP was performed among the GEP70-defined low-risk patients (Table 4). Unfortunately, small sample sizes in the baseline RC4 group hindered this analysis. We do note a significant increase in the hazard for progression among patients with baseline RC4 who improved to RC1–3 at transplant compared with patients with RC1–3 at both time points, showing that even when patients recover from RI before transplant, they still have worse outcome compared with patients with baseline RC1–3. There were trends for increased hazard of outcome (both PFS and TTP) for the baseline RC4 groups (with either RC1–3 or RC4 at the time of transplant) compared with the group with RC1–3 at both baseline and transplant. Of interest, among the subset of patients with improvement from RC4 to RC1–3 before transplant, the majority did not revert back to RC4 upon disease relapse (data not shown). The models were dominated by the well-recognized baseline prognostic variables (albumin, B2M, C-reactive protein, platelet count, IgA isotype and CA). The inclusion of bortezomib in TT3a and TT3b improved both PFS and TTP when other variables were accounted for. Achieving MM complete response status before transplant was the only beneficial post-treatment variable.

## Discussion

We validated the adverse prognostic consequences of RI as measured by eGFR values <30 ml/min/1.73 m^2^. The improvement in clinical outcomes over the course of TT2 and TT3 protocols was limited to patients lacking RC4. The failure of RC4 patients to derive benefit from bortezomib in TT3 trials is at variance with HOVON data.^13^ Baseline RC4 maintained its adverse impact in multivariate analyses among the subset of GEP70-defined low-risk patients, even if RC1–3 was achieved by the time of transplant. Based on the TTP analyses performed, this adverse impact appears to be MM-related, which is supported also by overrepresentation of prognostically adverse MM features (anemia; elevated levels of lactate dehydrogenase, C-reactive protein and bone marrow plasma cells; and presence of CA13 and/or hypodiploidy). Pursuing a MM-related RC4 prognostic feature, we identified 46 gene probes distinguished RC4 from RC1–3 classes which, however, did not appear to relate to MM biology or survival.

## Figures and Tables

**Table 1:** Baseline characteristics of patients enrolled in Total Therapy protocols TT2 (TT2*−*Thal; TT2+Thal) and TT3

*Factor*	*Combined*	*TT2 −Thal*	*TT2 +Thal*	*TT3a*	*TT3b*	P*-value*
Age ⩾65 years	266/1148 (23%)	72/345 (21%)	64/323 (20%)	84/303 (28%)	46/177 (26%)	0.062
Female	451/1148 (39%)	135/345 (39%)	137/323 (42%)	110/303 (36%)	69/177 (39%)	0.482
White	1035/1148 (90%)	309/345 (90%)	293/323 (91%)	270/303 (89%)	163/177 (92%)	0.704
**Albumin** **<3.5 g/dl**	**280/1144 (24%)**	**59/343 (17%)**	**60/321 (19%)**	**80/303 (26%)**	**81/177 (46%)**	**<0.001**
**B2M >5.5 mg/l**	**238/1146 (21%)**	**63/345 (18%)**	**59/323 (18%)**	**65/303 (21%)**	**51/175 (29%)**	**0.023**
Creatinine ⩽1.5 mg/dl	996/1148 (87%)	293/345 (85%)	279/323 (86%)	267/303 (88%)	157/177 (89%)	0.548
Creatinine >1.5 and ⩽2 mg/dl	67/1148 (6%)	20/345 (6%)	21/323 (7%)	14/303 (5%)	12/177 (7%)	0.706
Creatinine >2 mg/dl	85/1148 (7%)	32/345 (9%)	23/323 (7%)	22/303 (7%)	8/177 (5%)	0.245
CRP ⩾8 mg/l	422/1137 (37%)	127/339 (37%)	136/319 (43%)	100/302 (33%)	59/177 (33%)	0.063
BL RC4^a^	91/1148 (8%)	36/345 (10%)	24/323 (7%)	23/303 (8%)	8/177 (5%)	0.103
BL RC3^a^	235/1148 (20%)	68/345 (20%)	69/323 (21%)	60/303 (20%)	38/177 (21%)	0.925
BL RC2^a^	563/1148 (49%)	169/345 (49%)	157/323 (49%)	153/303 (50%)	84/177 (47%)	0.928
BL RC1^a^	259/1148 (23%)	72/345 (21%)	73/323 (23%)	67/303 (22%)	47/177 (27%)	0.539
Hb <10 g/dl	310/1147 (27%)	80/345 (23%)	81/322 (25%)	94/303 (31%)	55/177 (31%)	0.070
LDH ⩾190 U/l	328/1146 (29%)	98/344 (28%)	106/322 (33%)	81/303 (27%)	43/177 (24%)	0.168
Platelet Count <150 × 10^9^/l	172/1147 (15%)	54/345 (16%)	54/322 (17%)	38/303 (13%)	26/177 (15%)	0.493
IgG isotype	625/1148 (54%)	186/345 (54%)	177/323 (55%)	173/303 (57%)	89/177 (50%)	0.542
IgA isotype	271/1148 (24%)	77/345 (22%)	84/323 (26%)	72/303 (24%)	38/177 (21%)	0.617
**Bence Jones proteinuria**^b^	**528/1122 (47%)**	**155/339 (46%)**	**134/318 (42%)**	**141/300 (47%)**	**98/165 (59%)**	**0.004**
Light chain only	214/1148 (19%)	67/345 (19%)	51/323 (16%)	54/303 (18%)	42/177 (24%)	0.178
Non-secretory disease	22/1148 (2%)	9/345 (3%)	7/323 (2%)	2/303 (1%)	4/177 (2%)	0.222
Ratio of involved–uninvolved	*n*=570	*n*=64	*n*=62	*n*=279	*n*=165	0.107
(*n*, median (range))	82 (2–255556)	59 (2–18111)	73 (3–5160)	65 (2–142222)	146 (2–255555)	
Cytogenetic abnormalities	362/1127 (32%)	104/339 (31%)	93/322 (29%)	95/293 (32%)	70/173 (40%)	0.065
GEP 70 high risk	123/795 (15%)	20/176 (11%)	26/175 (15%)	40/276 (14%)	37/168 (22%)	0.052
GEP PR subgroup	111/795 (14%)	27/176 (15%)	24/175 (14%)	33/276 (12%)	27/168 (16%)	0.606

Abbreviations: B2M, β-2-microglobulin; BL, baseline; CRP, C-reactive protein; eGFR, estimated glomerular filteration rate; GEP, gene expression profiling; Hb, hemoglobin; Ig, immunoglobulin; LDH, lactate dehydrogenase; *n*, number with factor, *N*, number with valid data for factor; ND, no valid observations for factor; PR, proliferation; RC, renal classes; Thal, thalidomide. *P*-value is from chi-squared test for binary variables; *P*-value is from Kruskal–Wallis test for continuous variables.

a RC1 (eGFR ⩾90 ml/min/1.73 m^2^), RC2 (eGFR 60–89 ml/min/1.73 m^2^), RC3 (eGFR 30–59 ml/min/1.73 m^2^) and RC4 (eGFR <30 ml/min/1.73 m^2^).

b M protein is present in urine (regardless of heavy-chain or light-chain types). Bold is always associated with *P*-value <0.05.

**Table 2:** Univariate and multivariate Cox regression models for TT2 and TT3 protocols combined, examining RC4 compared with RC1-3

		*PFS*	
*Variable*	n/N *(%)*	*HR (95% CI)*	P*-value*
*A. All TT2 and TT3 patients, no GEP variables*			
Univariate			
**RC4**^a^	**91/1148 (8%)**	**1.73 (1.36, 2.21)**	**<0.001**
Multivariate			
RC4	83/1069 (8%)	1.04 (0.77, 1.41)	0.778
**Albumin <3.5 g/dl**	**259/1069 (24%)**	**1.35 (1.13, 1.62)**	**<0.001**
**B2M >5.5 mg/l**	**221/1069 (21%)**	**1.52 (1.22, 1.88)**	**<0.001**
**LDH** ⩾**190 U/l**	**313/1069 (29%)**	**1.28 (1.08, 1.51)**	**0.004**
**Platelet count <150 × 10**^**9**^**/l**	**161/1069 (15%)**	**1.43 (1.17, 1.75)**	**<0.001**
**IgA isotype**	**255/1069 (24%)**	**1.26 (1.06, 1.51)**	**0.009**
**Cytogenetic abnormalities**	**347/1069 (32%)**	**1.52 (1.29, 1.78)**	**<0.001**
**TT3 indicator**^b^	**446/1069 (42%)**	**0.61 (0.51, 0.72)**	**<0.001**
			
*B. All TT2 and TT3 patients, GEP70 risk variable*			
Univariate			
**RC4**^a^	**91/1148 (8%)**	**1.73 (1.36, 2.21)**	**<0.001**
Multivariate			
RC4	64/751 (9%)	1.10 (0.77, 1.56)	0.609
**B2M >5.5 mg/l**	**172/751 (23%)**	**1.52 (1.19, 1.96)**	**<0.001**
**LDH** ⩾**190 U/l**	**228/751 (30%)**	**1.29 (1.05, 1.59)**	**0.015**
**Platelet count <150 × 10**^**9**^**/l**	**116/751 (15%)**	**1.31 (1.02, 1.69)**	**0.032**
**Light chain only**	**142/751 (19%)**	**0.68 (0.53, 0.88)**	**0.004**
**Cytogenetic abnormalities**	**260/751 (35%)**	**1.37 (1.12, 1.68)**	**0.002**
**GEP 70 high risk**	**119/751 (16%)**	**1.94 (1.49, 2.52)**	**<0.001**
**TT3 indicator**^b^	**415/751 (55%)**	**0.59 (0.49, 0.72)**	**<0.001**
			
*C. TT2 and TT3 GEP70 low risk patients*			
Univariate			
**RC4**^a^	**42/672 (6%)**	**2.25 (1.58, 3.21)**	**<0.001**
Multivariate			
**RC4**	**42/632 (7%)**	**1.66 (1.07, 2.58)**	**0.025**
**B2M >5.5 mg/l**	**116/632 (18%)**	**1.57 (1.16, 2.11)**	**0.003**
**Platelet count <150 × 10**^**9**^**/l**	**75/632 (12%)**	**1.74 (1.30, 2.31)**	**<0.001**
**Light chain only**	**116/632 (18%)**	**0.69 (0.51, 0.93)**	**0.014**
**Cytogenetic abnormalities**	**173/632 (27%)**	**1.35 (1.08, 1.71)**	**0.010**
**TT3 indicator**^b^	**340/632 (54%)**	**0.57 (0.46, 0.71)**	**<0.001**

Abbreviations: B2M, β-2-microglobulin; CI, confidence interval; GEP, gene expression profiling; HR, hazard ratio; LDH, lactate dehydrogenase; NS2, multivariate results not statistically significant at 0.05 level; PFS, progression-free survival; RC, renal classes; TT, Total Therapy. *P*-value from Wald *χ*^2^-test in Cox-regression.

a Univariate HR and *P*-value are shown only for RC4 (vs RC1–3). RC4 was found to be univariately significant for all models considered, and was retained in the multivariate model for GEP70 low-risk patients.

b Variable to account for differences in baseline characteristics between TT3a/b (included bortezomib) and TT2 (did not include bortezomib); TT2±thalidomide is the reference group for this variable.

All univariate P-values reported regardless of significance. Multivariate model uses stepwise selection with entry level 0.1 and variable remains if meets the 0.05 level. A multivariate *P*-value >0.05 indicates variable forced into the model with significant variables chosen using stepwise selection. Bold is always associated with *P*-value <0.05.

**Table 3:** Logistic regression analysis of baseline variables linked to RC4

	*RC4 (vs RC1-3)*				
*Factor*	n	*With factor*	*Without factor*	*OR (95% CI)*	P*–value*
*Univariate*					
**Age** ⩾**65 years**	**1148**	**32/266 (12%)**	**59/882 (7%)**	**1.91 (1.21, 3.00)**	**0.005**
**Female**	**1148**	**47/451 (10%)**	**44/697 (6%)**	**1.73 (1.12, 2.65)**	**0.013**
White	1148	87/1035 (8%)	4/113 (4%)	2.50 (0.90, 6.94)	0.079
Albumin <3.5 g/dl	1144	30/280 (11%)	61/864 (7%)	1.58 (1.00, 2.50)	0.051
**CRP** ⩾**8 mg/l**	**1137**	**57/422 (14%)**	**33/715 (5%)**	**3.23 (2.06, 5.05)**	**<0.001**
**Hb <10 g/dl**	**1147**	**58/310 (19%)**	**33/837 (4%)**	**5.61 (3.57, 8.80)**	**<0.001**
**LDH** ⩾**190 U/l**	**1146**	**48/328 (15%)**	**43/818 (5%)**	**3.09 (2.00, 4.77)**	**<0.001**
**BMPC** ⩾**33%**	**1012**	**66/610 (11%)**	**14/402 (3%)**	**3.36 (1.86, 6.07)**	**<0.001**
Cytogenetic abnormalities	1127	36/362 (10%)	52/765 (7%)	1.51 (0.97, 2.36)	0.067
**CA13/hypo**	**1127**	**35/233 (15%)**	**53/894 (6%)**	**2.81 (1.78, 4.42)**	**<0.001**
**GEP 70 high risk**	**795**	**23/123 (19%)**	**42/672 (6%)**	**3.45 (1.99, 5.98)**	**<0.001**
**GEP 80 high risk**	**795**	**9/58 (16%)**	**56/737 (8%)**	**2.23 (1.04, 4.78)**	**0.038**
GEP CD-1 subgroup	795	2/58 (3%)	63/737 (9%)	0.38 (0.09, 1.60)	0.189
GEP CD-2 subgroup	795	11/112 (10%)	54/683 (8%)	1.27 (0.64, 2.51)	0.494
**GEP HY subgroup**	**795**	**9/244 (4%)**	**56/551 (10%)**	**0.34 (0.16, 0.70)**	**0.003**
GEP LB subgroup	795	9/110 (8%)	56/685 (8%)	1.00 (0.48, 2.09)	0.998
GEP MF subgroup	795	7/53 (13%)	58/742 (8%)	1.79 (0.78, 4.15)	0.172
GEP MS subgroup	795	7/107 (7%)	58/688 (8%)	0.76 (0.34, 1.71)	0.509
**GEP PR subgroup**	**795**	**20/111 (18%)**	**45/684 (7%)**	**3.12 (1.76, 5.52)**	**<0.001**
**GEP proliferation index ⩾10**	**795**	**15/85 (18%)**	**50/710 (7%)**	**2.83 (1.51, 5.30)**	**0.001**
GEP centrosome index ⩾3	795	15/189 (8%)	50/606 (8%)	0.96 (0.53, 1.75)	0.890
					
*Multivariate*					
**CRP** ⩾**8 mg/l**	**726**	**35/254 (14%)**	**23/472 (5%)**	**2.58 (1.44, 4.60)**	**0.001**
**Hb <10 g/dl**	**726**	**36/209 (17%)**	**22/517 (4%)**	**3.21 (1.78, 5.78)**	**<0.001**
**LDH** ⩾**190 U/l**	**726**	**33/213 (15%)**	**25/513 (5%)**	**2.45 (1.38, 4.38)**	**0.002**
**BMPC** ⩾**33%**	**726**	**51/470 (11%)**	**7/256 (3%)**	**3.07 (1.33, 7.09)**	**0.009**

Abbreviations: BMPC, bone marrow plasmacytosis; CA, cytogenetic abnormalities; CD, cyclin D (GEP subgroup); CI, confidence interval; CRP, C-reactive protein; Hb, hemoglobin; HY, hyperdiploidy; LB, low bone; LDH, lactate dehydrogenase; MF, mammalian factor; MS, MMSET; NS2, multivariate results not statistically significant at 0.05 level; OR, odds ratio; PR, proliferation; RC, renal classes. *P*-value from Wald *χ*^2^-test in logistic regression.

Univariate *P*-values reported regardless of significance. Multivariate model uses stepwise selection with entry level 0.1 and variable remains if meets the 0.05 level. A multivariate *P*-value >0.05 indicates variable forced into model with significant variables chosen using stepwise selection. Bold is always associated with *P*-value <0.05.

**Table 4:** Multivariate analysis of baseline variables, RC both at baseline and prior to transplant, and myeloma CR affecting PFS and TTP

*TT2 and TT3 GEP70 low-risk patients*		*Progression-free survival* *from first transplant*		*Time to progression* *from first transplant*	
*Variable*	n/N *(%)*	*HR (95% CI)*	P*-value*	*HR (95% CI)*	P*-value*
BL RC1-3, TX1 RC1-3^a^	536/570 (94%)	(Reference)	(Reference)	(Reference)	(Reference)
**BL RC 4, TX1 RC1-3**^a^	**20/570 (4%)**	1.42 (0.77, 2.60)	0.261	**2.21 (1.14, 4.27)**	**0.019**
BL RC4, TX1 RC4^a^	14/570 (2%)	1.55 (0.81, 2.99)	0.190	1.89 (0.82, 4.37)	0.137
**Albumin <3.5 g/dl**	**122/570 (21%)**	NS	NS	**1.66 (1.21, 2.28)**	**0.002**
**B2M >5.5 mg/l**	**90/570 (16%)**	**1.54 (1.08, 2.18)**	**0.016**	NS	NS
**CRP** ⩾**8 mg/l**	**185/570 (32%)**	NS	NS	**0.71 (0.52, 0.97)**	**0.029**
**Platelet count <150 × 10**^**9**^**/l**	**64/570 (11%)**	**1.86 (1.35, 2.55)**	**<0.001**	**1.99 (1.38, 2.85)**	**<0.001**
**IgA**	**135/570 (24%)**	NS	NS	**1.43 (1.06, 1.93)**	**0.019**
**Cytogenetic abnormalities**	**156/570 (27%)**	**1.53 (1.19, 1.95)**	**<0.001**	**1.37 (1.02, 1.85)**	**0.035**
**Indicator of protocol with bortezomib (TT3a, TT3b)**^b^	**317/570 (57%)**	**0.54 (0.43, 0.69)**	**<0.001**	**0.38 (0.29, 0.51)**	**<0.001**
**MM CR achieved prior to transplant**	**71/570 (12%)**	**0.34 (0.22, 0.54)**	**<0.001**	**0.37 (0.22, 0.63)**	**<0.001**

Abbreviations: B2M, β-2-microglobulin; BL, baseline; CI, confidence interval; CR, complete response; CRP, C-reactive protein; eGFR, estimated glomerular filteration rate; GEP, gene expression profiling; Hb, hemoglobin; HR, hazard ratio; Ig, immunoglobulin; LDH, lactate dehydrogenase; MM, multiple myeloma; NS, not significant; PFS, progression-free survival; RC, renal classes; TT, Total Therapy; TTP, time to progression; TTX1, transplant 1. *P*-value from Wald *χ*^2^-test in Cox regression.

a RC1 (eGFR ⩾90 ml/min/1.73 m^2^), RC2 (eGFR 60–89 ml/min/1.73 m^2^), RC3 (eGFR 30–59 ml/min/1.73 m^2^), and RC4 (eGFR <30 ml/min/1.73 m^2^).

b Variable to account for differences in baseline characteristics between TT3a/b (included bortezomib) and TT2 (did not include bortezomib); TT2±thalidomide is the reference group for this variable.

The BL RC1–3 and TX1 RC4 group was excluded from this analysis due to exceptionally small sample size, *n*=5.

Variables considered for multivariate analysis (GEP70 low-risk patients only) were: albumin <3.5 g/dl, B2M >5.5 mg/l, CRP⩾8 mg/l, Baseline—PreTX1 eGFR group, Hb<10 g/dl, LDH⩾190 U/l, platelet count <150 × 10^9^/l, IgA isotype, light chain only, non-secretory disease, kappa light chain, urine M protein present, cytogenetic abnormalities, indicator of protocol with bortezomib, MM CR achieved before transplant. Multivariate model uses stepwise selection with entry level 0.1 and variable remains if meets the 0.05 level. A multivariate *P*-value >0.05 indicates variable forced into model with significant variables chosen using stepwise selection. Indicator of bortezomib use in protocol (TT3a, TT3b), CR achieved before TX1. Bold is always associated with *P*-value <0.05.

## Notes

Dr Barlogie has received research funding from Celgene and Millennium, is a consultant to Celgene and Millennium, and is a co-inventor on patents and patent applications related to use of GEP in cancer medicine, that have been licensed to Myeloma Health, LLC.
