# Supplementary material for: Renal insufficiency retains adverse prognostic implications despite renal function improvement following Total Therapy for newly diagnosed multiple myeloma
Source: Leukemia. 2015 Mar 13;29(5):1195–201. doi: 10.1038/leu.2015.15 (PMC4430702; doi:10.1038/leu.2015.15)
Supplement: Supplementary Tables [file leu201515x1.docx]

**Supplemental Tables**

**Table 1A: Number of significant probes by cut-off, TT2+3 GEP-70 low-risk patients**

|  | 0.0001 | 0.001 | 0.01 | 0.025 | **0.05** | 0.1 |
| --- | --- | --- | --- | --- | --- | --- |
| p-value | 75 | 398 | 1862 | 3549 | **5708** | 9432 |
| q-value | 0 | 1 | 6 | 27 | **46** | 260 |

**Table 1B: List of 46 significant probes at q<0.05**

| probe | gene_symbol | chrom_loc | mean.eGFRgt30 | mean.eGFRlte30 | fold.chg | q |
| --- | --- | --- | --- | --- | --- | --- |
| 225045_at | CCDC88A | chr2p16.1 | 9.027818 | 10.18961 | 2.237358 | 0.000526 |
| 210237_at | ARTN | chr1p33-p32 | 7.155662 | 6.44061 | 1.641543 | 0.002717 |
| 228567_at | LOC100996430 /// MIR4720 | --- | 8.438325 | 8.747683 | 1.239156 | 0.002717 |
| 210773_s_at | FPR2 | chr19q13.3-q13.4 | 6.365475 | 7.010735 | 1.564021 | 0.005307 |
| 238822_at | --- | --- | 7.367119 | 7.910843 | 1.45773 | 0.009049 |
| 227639_at | PIGK | chr1p31.1 | 10.59107 | 11.09017 | 1.41333 | 0.009307 |
| 222576_s_at | AGO1 | chr1p34.3 | 8.485334 | 8.854807 | 1.291881 | 0.010809 |
| 217900_at | IARS2 | chr1q41 | 11.53433 | 11.90227 | 1.290511 | 0.012426 |
| 219329_s_at | ATRAID | chr2p23.3 | 13.38302 | 13.77775 | 1.314693 | 0.012426 |
| 225424_at | GPAM | chr10q25.2 | 7.932965 | 8.379241 | 1.362518 | 0.012426 |
| 1559240_at | LOC100507053 | --- | 6.597002 | 7.175289 | 1.493075 | 0.012444 |
| 202353_s_at | PSMD12 | chr17q24.2 | 10.21801 | 10.66356 | 1.361827 | 0.012694 |
| 225113_at | AGPS | chr2q31.2 | 7.746244 | 8.338451 | 1.50755 | 0.013445 |
| 222208_s_at | POLR2J4 | chr7p13 | 7.645006 | 8.12707 | 1.39674 | 0.015673 |
| 235892_at | --- | --- | 7.093759 | 7.598 | 1.418377 | 0.017328 |
| 216942_s_at | CD58 | chr1p13 | 7.968406 | 8.632016 | 1.584042 | 0.01842 |
| 218170_at | ISOC1 | chr5q22.1-q33.3 | 11.80887 | 12.17597 | 1.289756 | 0.01842 |
| 228039_at | DDX46 | chr5q31.1 | 9.251208 | 9.585511 | 1.260768 | 0.01842 |
| 241858_at | TNNI3K | chr1p31.1 | 5.884336 | 6.412549 | 1.442141 | 0.01842 |
| 225108_at | AGPS | chr2q31.2 | 8.457264 | 8.844973 | 1.308314 | 0.021587 |
| 206760_s_at | FCER2 | chr19p13.3 | 5.437997 | 4.677816 | 1.693702 | 0.021877 |
| 212548_s_at | FRYL | chr4p11 | 10.17615 | 10.50342 | 1.254639 | 0.021877 |
| 213803_at | KPNB1 | --- | 9.621947 | 9.966789 | 1.270012 | 0.021877 |
| 214930_at | SLITRK5 | chr13q31.2 | 5.528344 | 4.453029 | 2.107182 | 0.021877 |
| 227531_at | CLOCK | chr4q12 | 10.25486 | 10.61516 | 1.283688 | 0.021877 |
| 243539_at | KIAA1841 | chr2q14 | 5.9465 | 6.81865 | 1.830389 | 0.021877 |
| 202470_s_at | CPSF6 | chr12q15 | 7.296549 | 7.898175 | 1.517426 | 0.024164 |
| 205292_s_at | HNRNPA2B1 | chr7p15 | 13.20721 | 13.43621 | 1.172022 | 0.026134 |
| 217672_x_at | EIF1 | chr17q21.2 | 9.304121 | 9.618358 | 1.243354 | 0.028266 |
| 218924_s_at | CTBS | chr1p22 | 10.65272 | 11.16428 | 1.425588 | 0.028266 |
| 236231_at | ZNF271 | chr18q12 | 6.514591 | 6.901727 | 1.307795 | 0.028266 |
| 201545_s_at | BCL2L2-PABPN1 /// PABPN1 | chr14q /// chr14q11.2 | 10.03487 | 10.31253 | 1.212229 | 0.0289 |
| 226230_at | SMEK2 | chr2p16.1 | 10.53817 | 10.79742 | 1.196857 | 0.0289 |
| 226561_at | AGFG1 | chr2q36.3 | 8.803106 | 9.246153 | 1.359473 | 0.029464 |
| 201589_at | SMC1A | chrXp11.22-p11.21 | 10.98742 | 11.29944 | 1.241442 | 0.032415 |
| 211882_x_at | FUT6 | chr19p13.3 | 5.427928 | 4.920765 | 1.421252 | 0.032415 |
| 225350_s_at | ZYG11B | chr1p32.3 | 10.55974 | 10.7791 | 1.164211 | 0.032415 |
| 225497_at | ATE1 | chr10q26.13 | 10.2348 | 10.62761 | 1.312945 | 0.032415 |
| 235531_at | --- | --- | 8.448052 | 8.79676 | 1.27342 | 0.032415 |
| 1558019_at | --- | --- | 6.174948 | 7.080857 | 1.873726 | 0.032498 |
| 213573_at | KPNB1 | --- | 9.633192 | 9.990486 | 1.28102 | 0.035853 |
| 225976_at | BTF3L4 | chr1p32.3 | 11.05121 | 11.28384 | 1.174977 | 0.040376 |
| 228919_at | --- | --- | 7.070694 | 6.346291 | 1.652217 | 0.043936 |
| 208498_s_at | ACTG1P4 /// AMY1A /// AMY1B /// AMY1C /// AMY2A /// AMY2B | chr1p21 /// chr1p21.1 | 7.121676 | 7.969134 | 1.799327 | 0.04537 |
| 225535_s_at | TIMM23 /// TIMM23B | chr10q11.23 | 11.57772 | 11.8321 | 1.19283 | 0.047363 |
| 218724_s_at | TGIF2 | chr20q11.23 | 8.775323 | 8.38534 | 1.310378 | 0.049248 |
